# Predictive value of tumor mutation burden (TMB) with targeted next-generation sequencing in immunocheckpoint inhibitors for non-small cell lung cancer (NSCLC)

**DOI:** 10.7150/jca.48105

**Published:** 2021-01-01

**Authors:** Xiaoting Ma, Yujian Zhang, Shan Wang, Jing Yu

**Affiliations:** Cancer Center, Beijing Friendship Hospital, Capital Medical University, No. 95 Yong An Road, Xi Cheng District, Beijing, 100050, China.

**Keywords:** disease control rate, immune checkpoint inhibitor, NSCLC, objective response rate, overall survival, progression-free survival, tumor mutation burden

## Abstract

**Background:** To evaluate the clinical predictive value of tumor mutation burden (TMB) for immune checkpoint inhibitor (ICI) therapy in patients with non-small cell lung cancer (NSCLC).

**Method:** As of 15 February 2020, PubMed, PMC and EMBASE databases as well as the American society of clinical oncology (ASCO) and European society of medical oncology (ESMO) databases were searched. The Mantel-Haenszel or inverse variance weighted fixed-effects model (I^2^ ≤ 50%) or random-effects model (I^2^ > 50%) were used to evaluate OR and its 95% CI of objective response rate (ORR) and disease control rate (DCR) , as well as HR and its 95% CI of progression-free survival (PFS) and overall survival (OS). In addition, we did publication bias, heterogeneity analysis, sensitivity analysis and subgroup analysis. And quality of the studies included and the level of evidence for outcome measures were evaluated.

**Results:** 14 studies involving 2872 patients were included. The ORR (OR 3.52, 95%CI 2.32-5.35, *p* < 0.00001), DCR (OR 3.26, 95%CI 1.91-5.55, *p* < 0.0001), PFS (HR 0.81, 95%CI 0.74-0.89, *p* < 0.00001) and OS (HR 0.83, 95%CI 0.74-0.94, *p* = 0.002) of ICI therapy in the high TMB group were all superior to those in the low TMB group.

**Conclusions:** TMB is a promising biomarker, which can predict the efficacy of ICI therapy in advanced NSCLC patients, included ORR, DCR, PFS and OS.

## Introduction

Immune checkpoint inhibitor (ICI) therapy, including immunomonoclonal antibodies against cytotoxic T lymphocyte-associated protein 4 (CTLA4), programmed cell death 1 (PD-1), or programmed death ligand 1 (PD-L1) antibodies, is a monoclonal antibody that negatively regulates T cell function 1. ICI has been approved for the therapy of many different types of cancer, including non-small cell lung cancer (NSCLC), melanoma, renal cell cancer, bladder cancer, and head and neck squamous cell carcinoma 1. Recently more and more predictive biomarkers have been used in immunotherapy research, such as tumor mutation burden (TMB), programmed cell death ligand 1 (PD-L1), and mismatch repair defect (dMMR)/microsatellites Instability (MSI) 2,3. Currently, PD-L1 and dMMR/MSI have been approved for clinical use 4. TMB refers to the total number of substitution and insertion/deletion mutations per megabase in the exon coding region of the gene being evaluated in the tumor cell genome 5. Although it has not yet been approved for clinical use, there is much evidence to support the clinical predictive value of TMB. First, tumor cells are genetically unstable and have high levels of somatic mutations, which may generate many new antigens, which activate T lymphocyte proliferation and kill tumor cells 6. In addition, the increase in neoantigen production is expected to make tumor cells more immunogenic, thereby increasing their response to immunotherapy 4. Studies have shown that TMB has many advantages over other biomarkers. First, it can be obtained in the blood, which may be an advantage in cases where tumor tissue specimens cannot be obtained 7. Second, compared to PD-L1 which can only predict the response of PD-1 / PD-L1 inhibitors, TMB can predict the response of multiple immunotherapies, including PD-1 / PD-L1 inhibitors, anti-CTLA4 antibodies (such as ipilimumab) and adoptive cell transfer therapy 8,9,10.

The incidence of somatic mutations in different types of tumors varies widely, with NSCLC having the highest mutation frequency, ranging from 0.1 to 100 mut/Mb 11. Through retrospective analysis, Rizvi 12 found that the efficacy of NSCLC patients for ICI therapy was related to the TMB threshold. NSCLC patients with high TMB thresholds had better efficacy and higher survival rates for NSCLC patients with low TMB. Klempner 13 also found that the TMB cut-off value of 10 mut/Mb can predict the efficacy of ICI in patients with NSCLC, and patients with higher TMB thresholds have longer progression-free survival (PFS). Most NSCLC studies using targeted Next Generation Sequencing (NGS) indicate that the cut-off value of TMB fluctuates around 10 mut/Mb.

Although many studies have revealed the predictive value of TMB for ICI therapy in NSCLC patients, some studies have reported negative results, especially in terms of long-term survival. This may be related to the fact that TMB has not received widespread attention and related research is less. In addition, the study population and immunotherapy regimens included in related studies are different, which may lead to different prediction results. In this study, we systematically reviewed and analyzed the relevant literature on the predictive value of TMB in NSCLC immunotherapy, in order to summarize the predictive effect of TMB on ICI therapy in patients with NSCLC.

## Methods

### Search strategy

**Figure 1:**
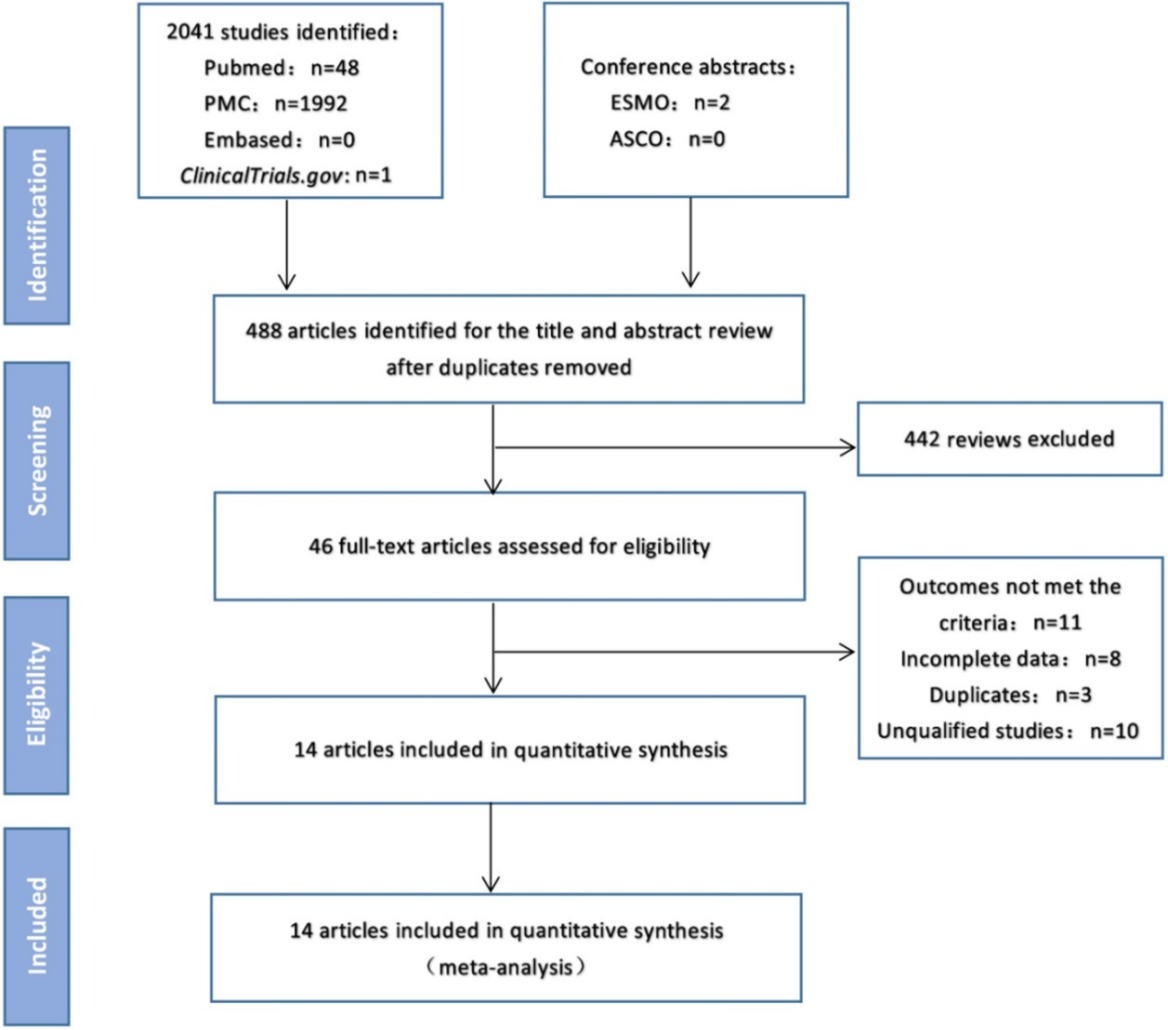
Study flow diagram.

As of February 15, 2020, electronic searches were performed on PubMed, PMC and EMBASE databases, as well as the American Society of Clinical Oncology (ASCO) and European Medical Oncology (ESMO) databases. The detailed search strategy is shown in Figure 1.The search term was as follows: (PD-1 OR PD-L1 OR CTLA-4 OR Ipilimumab OR Tremelimumab OR Nivolumab OR Pembrolizumab OR Lambrolizumab OR Atezolizumab OR Avelumab OR Durvalumab OR “immune checkpoint inhibitor” OR “immune checkpoint inhibitors” OR “ICI” OR “ICIs” OR “immune checkpoint blocker” OR “immune checkpoint blockers” OR “ICB” OR “ICBs”) AND (mutation burden OR mutational burden OR mutation load OR mutational load OR TMB OR TML) AND (“Next-Generation Sequencing” OR “Next Generation Sequencing” OR “Sequencing, Next-Generation”) AND (“Non-Small Cell Lung Cancer” OR “Non-Small Cell Lung Cancer” OR “NSCLC” OR “Carcinoma, Non-Small Cell Lung” OR “Non-Small Cell Lung Carcinoma” OR “Non-Small-Cell Lung Carcinoma” OR “Non-Small-Cell Lung Carcinomas” OR “Lung Carcinoma, Non-Small-Cell” OR “Lung Carcinomas, Non-Small-Cell” OR “Carcinoma, Non-Small Cell Lung” OR “Carcinomas, Non-Small-Cell Lung”). We searched all potentially relevant studies and reviewed the references in the final included articles to find possible missing studies.

### Inclusion criteria

The included studies should meet the following inclusion criteria: (1) Studies include TMB testing, the correlation analysis between TMB and efficacy evaluation of therapy of NSCLC patients with ICI (anti-CTLA-4, anti-PD-1, anti-PD-L1), and use the targeted NGS to calculate the cut-off value of TMB; (2) Evaluation indicators include the number of patients who achieved the objective response rate (ORR) and disease control rate (DCR) in the high TMB group and the low TMB group or hazard ratios (HR) and their 95% confidence intervals (95% CI) of progression-free survival (PFS) and overall survival (OS) are given in the article; (3) The number of people achieving ORR is the sum of complete response (CR) and partial response (PR), and the number of people achieving DCR is the sum of CR, PR and stable disease (SD).

### Data extraction

Two independent researchers extracted data from the included studies based on the preferred report project (PRISMA) for systematic evaluation and meta-analysis. All inconsistencies were resolved with the unanimous consent of all researchers. Information collected from these studies includes title, first author, publication year, number of patients, region, immunotherapy protocol, assessable TMB sample size, TMB cut-off value, number of people achieving ORR and DCR, HR and 95% CI of PFS and OS.

### Quality assessment

The Newcastle ottawa scale (NOS) was used to assess the quality of all included studies. The overall score ranges from 0 to 9, with 8-9 indicating high quality, 5-7 indicating medium quality, and studies below 5 indicate poor quality. GRADE is used to assess the level of evidence for all analysis results, which is classified as high quality, medium quality, low quality and very low quality.

### Statistical analysis

Statistical analysis was performed using Review Manager 5.3 and Forest plots were made. The main end point of the meta-analysis was to compare the ICI efficiency between the high TMB group and the low TMB group, and the evaluation indicators were OR and its 95% CI of ORR and DCR, and HR and its 95% CI of PFS and OS. State 12.0 was used to evaluate publication bias based on Begg's and Egger's tests. Heterogeneity between studies is represented by Cochrane's X^2^ statistics and the inconsistency statistic (I^2^). We consider I^2^ < 50% as low-level heterogeneity and I^2^ > 50% as significant heterogeneity. When I^2^ < 50%, the fixed effect model was used. When I^2^ > is 50%, the random effects model is used. In addition, we used sensitivity analysis to test the stability of the results. In order to further explore the predictive value of TMB on the effect of immunotherapy, subgroup analysis was performed by region and immunotherapy protocol. In all included studies, *p* < 0.05 was considered statistically significant.

## Results

### Characteristics of the included studies

Figure 1 shows the flow chart of this study. A total of 2043 records were retrieved through a database search. After excluding duplicate articles, 488 articles were eligible for inclusion and 442 reviews were deleted. The full text of the remaining 46 articles was then reviewed, and 14 studies 7,14-26 involving 2872 patients were finally included in the meta-analysis (Table 1). In these studies, 12 studies were for patients in western countries, 2 studies were for patients in Asian countries. And 10 studies were treated with anti-PD-1/PD-L1 monotherapy, 4 cohorts were treated with anti-PD-1/anti-PD-L1 combined with anti-CTLA-4 therapy.

### Quality assessment of included studies

NOS results indicate that 5 studies were high quality and 9 studies were medium quality, which ensures the relatively high quality of the study and improves the reliability of the meta-analysis (**Table 2**).

### Overall response rate (ORR) and disease control rate (DCR)

**Figure 2:**
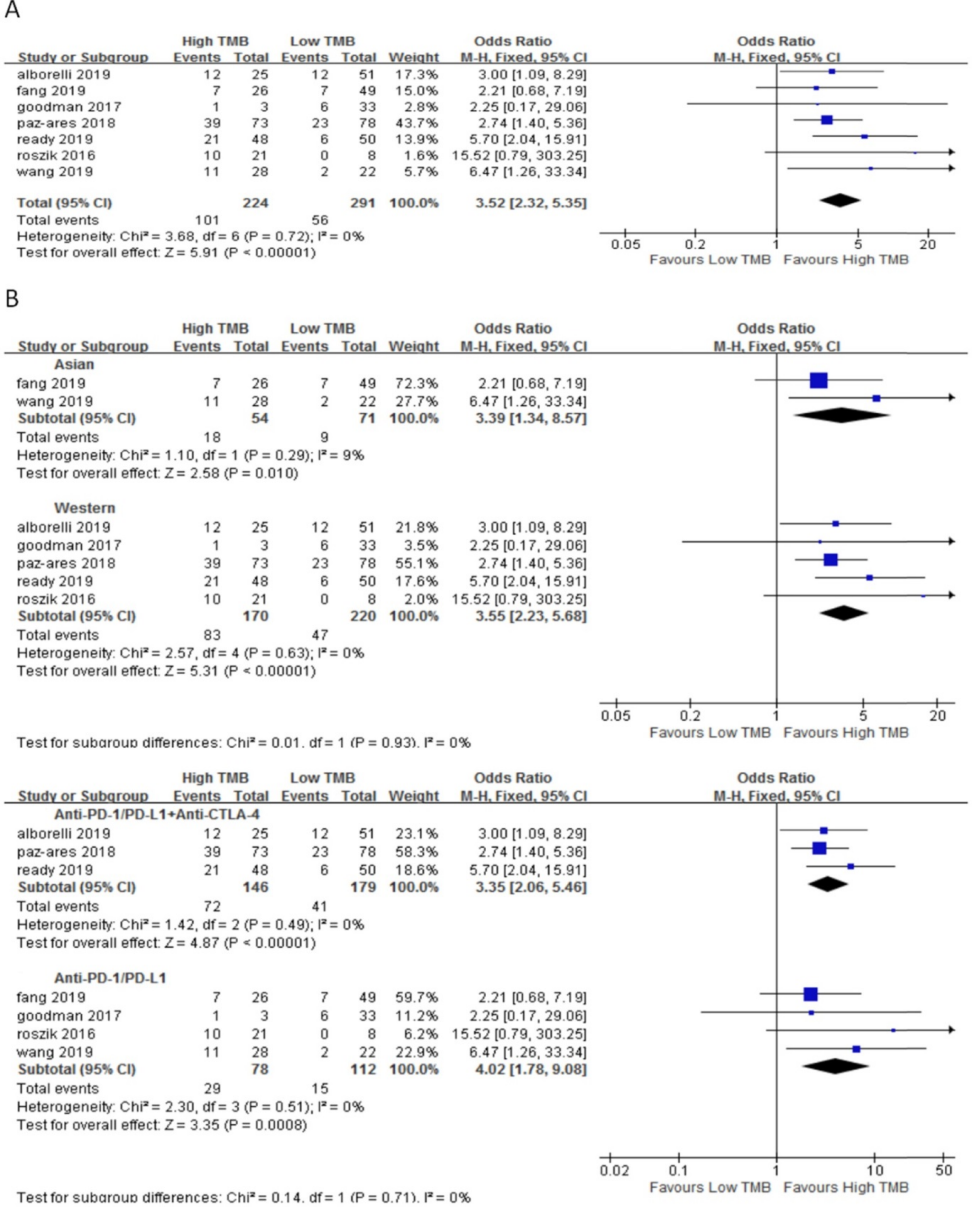
(A) Forest plot of association between TMB and ORR of immune checkpoint inhibitors. (B) Forest plot and pooled OR and 95% CI for subgroup ORR. TMB, tumor mutation burden; ORR, objective response rate; OR, odds ratio; CI, confidence interval.

In 7 studies including 515 NSCLC patients, we evaluated the relationship between TMB and ORR for ICI therapy. Compared with the low TMB group, the ORR of ICI therapy was significantly higher in the high TMB group (OR 3.52, 95%CI 2.32-5.35, *p* < 0.00001), and there was no heterogeneity (I^2^ = 0%, P = 0.72) (Fig. 2A). Subgroup analysis showed (Fig. 2B), in both Asian (OR 3.39, 95%CI 1.34-8.57,* p* = 0.01) and western (OR 3.55, 95%CI 2.23-5.68, *p* < 0.00001) groups, the ORR of patients with ICI therapy in the high TMB group was higher than those in the low TMB group. In the groups of anti-PD-1/anti-PD-L1 therapy (OR 4.02, 95%CI 1.78-9.08, *p* = 0.0008) and anti-PD-1/anti-PD-L1 combined with anti-CTLA-4 therapy (OR 3.35, 95%CI 2.06-5.46, *p* < 0.00001), ORR for ICI therapy was higher for patients in the high TMB group than those in the low TMB group.

**Figure 3:**
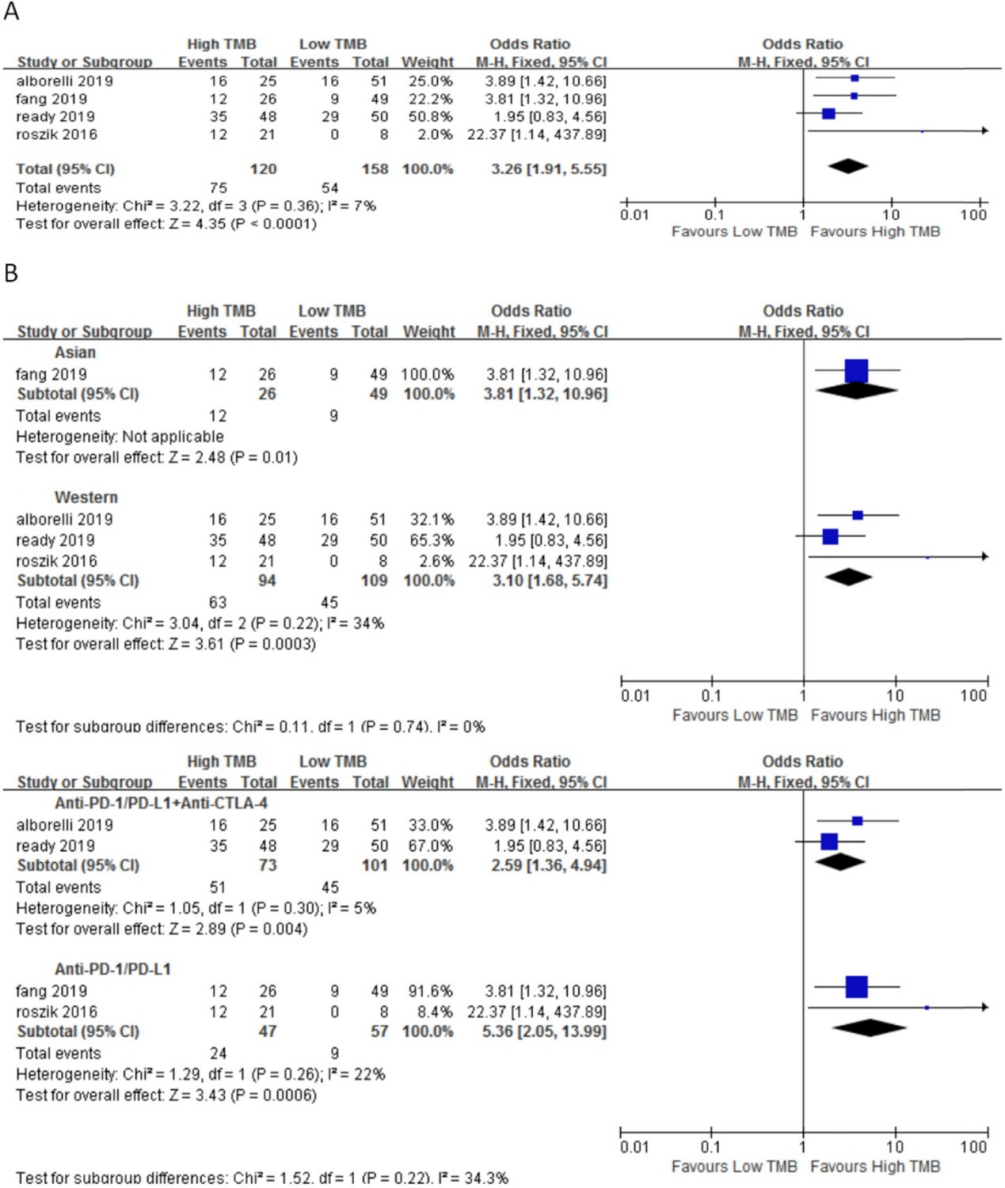
(A) Forest plot of association between TMB and DCR of immune checkpoint inhibitors. (B) Forest plot and pooled OR and 95% CI for subgroup DCR. TMB, tumor mutation burden; DCR, disease control rate; OR, odds ratio; CI, confidence interval.

In 4 studies involving 278 NSCLC patients, we evaluated the relationship between TMB and DCR for ICI therapy. DCR for ICI therapy in the high TMB group was significantly higher than that in the low TMB group (OR 3.26, 95%CI 1.91-5.55, *p* < 0.0001), and there was no heterogeneity (I^2^ = 7%, P = 0.36) (Fig. 3A). According to the subgroup analysis (Fig. 3B), in both Asian (OR 3.81, 95%CI 1.32-10.65, *p* = 0.01) and western (OR 3.10, 95%CI 1.68-5.74, *p* = 0.0003) groups, DCR for ICI therapy of patients in the high TMB group was higher than those in the low TMB group. In the groups of anti-PD-1/anti-PD-L1 therapy (OR 5.36, 95%CI 2.05-13.99, *p* = 0.0006) and anti-PD-1/anti-PD-L1 combined with anti-CTLA-4 therapy (OR 2.59, 95%CI 1.36-4.94, *p* = 0.004), DCR for ICI therapy was higher for patients in the high TMB group than those in the low TMB group.

### Overall survival (OS) and progression-free survival (PFS)

**Figure 4:**
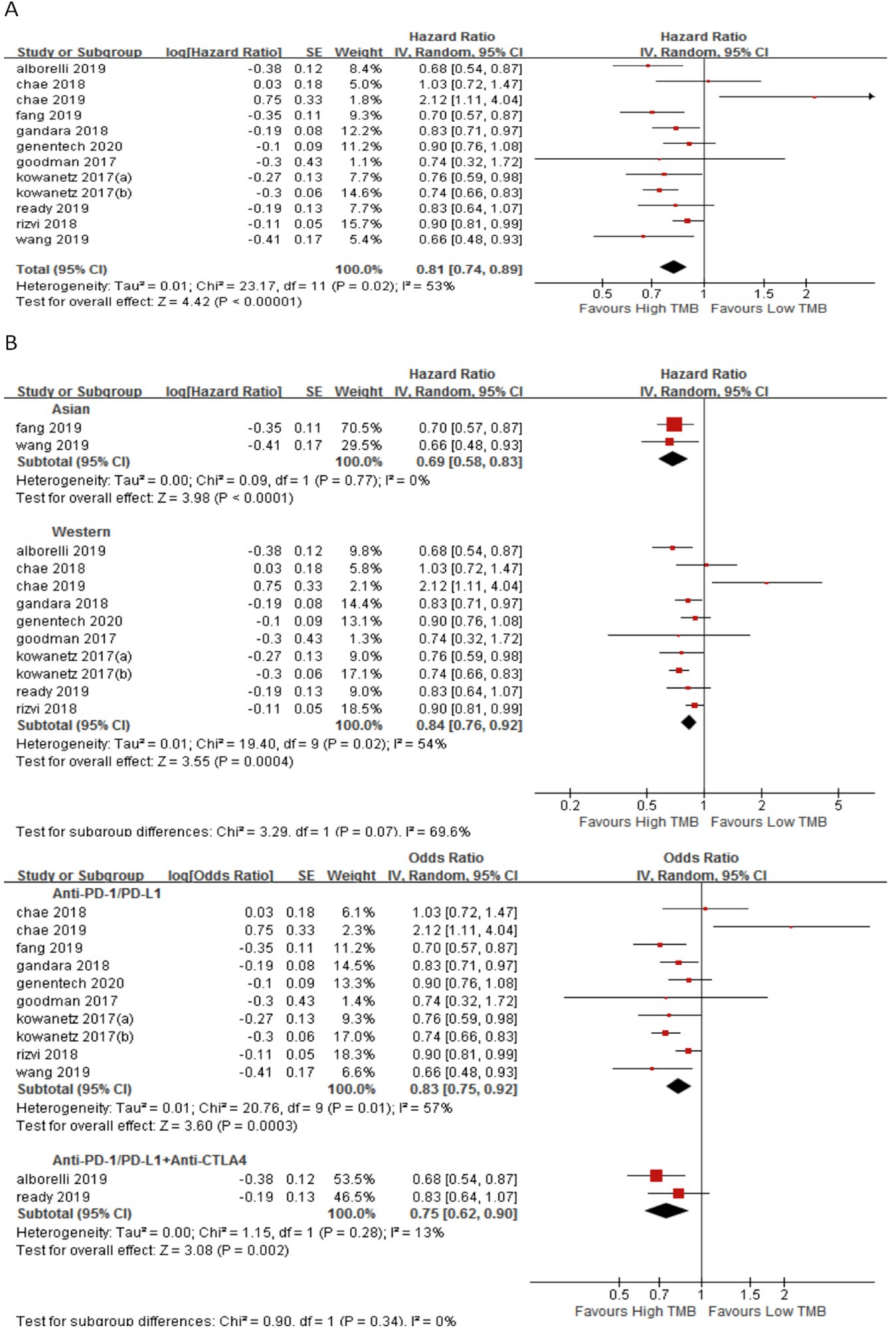
(A) Forest plot of association between TMB and PFS of immune checkpoint inhibitors. (B) Forest plot and pooled HR and 95% CI for subgroup PFS. TMB, tumor mutation burden; PFS, progression-free survival; HR, hazard ratio; CI, confidence interval.

In 11 studies involving 1688 NSCLC patients, we evaluated the relationship between TMB and PFS for ICI therapy. PFS for ICI therapy in the high TMB group was significantly better than that in the low TMB group (HR 0.81, 95%CI 0.74-0.89, *p* < 0.00001), and there was significant heterogeneity (I^2^ = 53%, *p* = 0.02) (Fig. 4A). Therefore, we performed a sensitivity analysis. After excluding the Goodman's study with the smallest weight (1.1%) and the Rizvi's study with the largest weight (15.7%), heterogeneity was unchanged and statistical result was stable. To further explore the sources of heterogeneity, we performed a subgroup analysis (Fig. 4B). In the Asian group, the PFS for ICI therapy of patients in the high TMB group was superior to that in the low TMB group (HR 0.69, 95%CI 0.58-0.83, *p* < 0.0001), while in the western group, there was no difference between the high TMB group and the low TMB group (HR 0.84, 95%CI 0.76-0.92, *p* = 0.0004). In the groups of anti-PD-1/anti-PD-L1 therapy (HR 0.83, 95%CI 0.75-0.92, *p* = 0.0003) and anti-PD-1/anti-PD-L1 combined with anti-CTLA-4 therapy (HR 0.75, 95%CI 0.62-0.90, *p* = 0.002), the PFS for ICI therapy was superior for patients in the high TMB group than that in the low TMB group.

**Figure 5:**
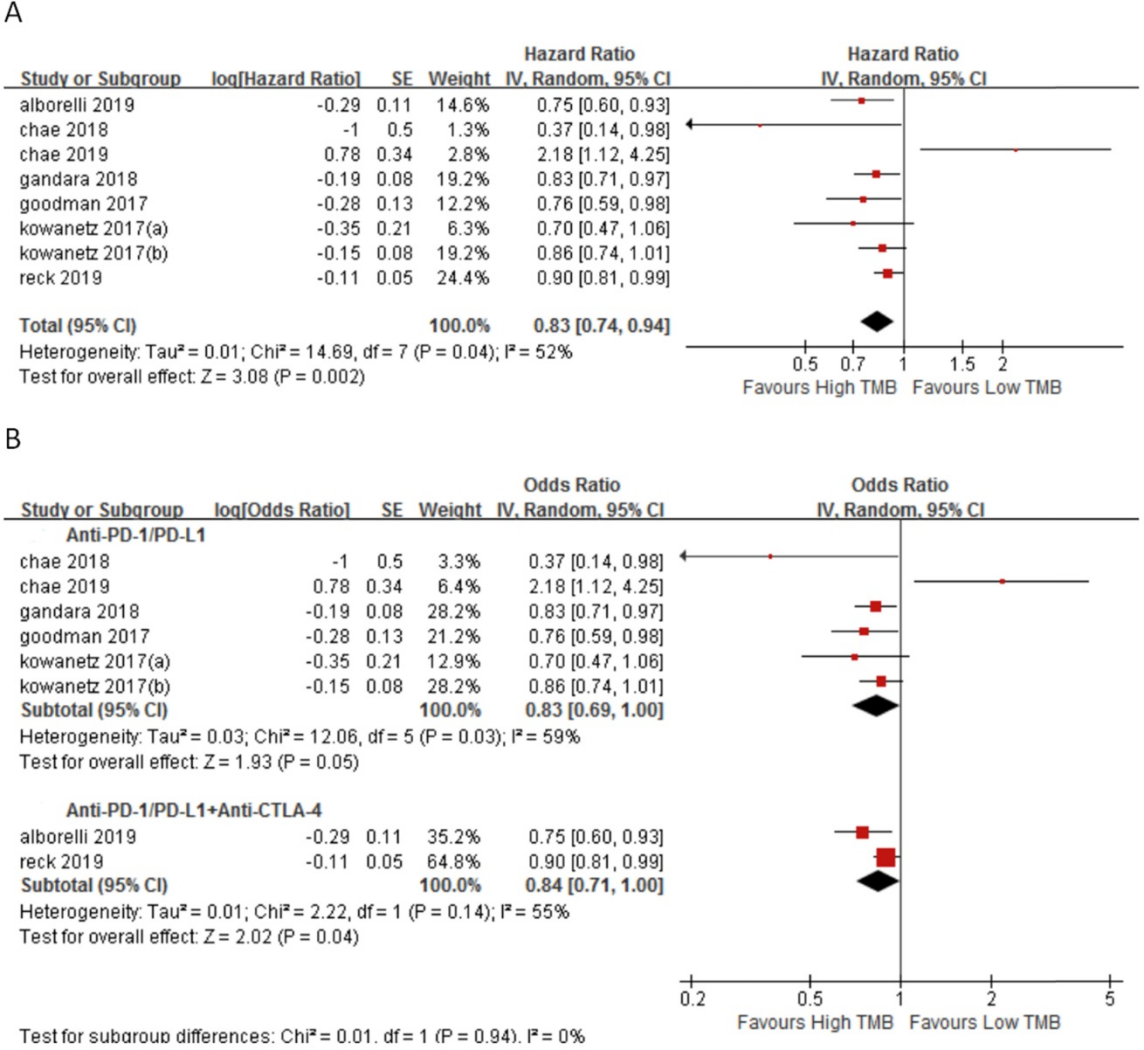
(A) Forest plot of association between TMB and OS of immune checkpoint inhibitors. (B) Forest plot and pooled HR and 95% CI for subgroup OS. TMB, tumor mutation burden; OS, overall survival; HR, hazard ratio; CI, confidence interval.

In 7 studies involving 2110 NSCLC patients, we evaluated the relationship between TMB and OS for ICI therapy. OS for ICI therapy in the high TMB group was significantly better than that in the low TMB group (HR 0.83, 95%CI 0.74-0.94, *p* = 0.002), and there was significant heterogeneity (I^2^ = 52%, *p* = 0.04) (Fig. 5A). We then performed a sensitivity analysis. After excluding the Reck's study with the largest weight (24.4%) and Chae's study with the smallest weight (1.3%), there was no significant change in the statistical result. To further explore the sources of heterogeneity, we performed a subgroup analysis (Fig. 5B). In the anti-PD-1/anti-PD-L1 combined with anti-CTLA-4 therapy group, the OS for ICI therapy of patients in the high TMB group was superior to that in the low TMB group (HR 0.84, 95%CI 0.71-1.00, *p* = 0.04), while in the anti-PD-1/anti-PD-L1 therapy group, there was no difference between the high TMB group and the low TMB group (HR 0.83, 95%CI 0.60-1.00, *p* = 0.05).

### Publication bias

After evaluation by Funnel plot, Egger's test and Begg's test, there was no evidence of publication bias (*p* > 0.05).

### Evidence level

**Figure 6:**
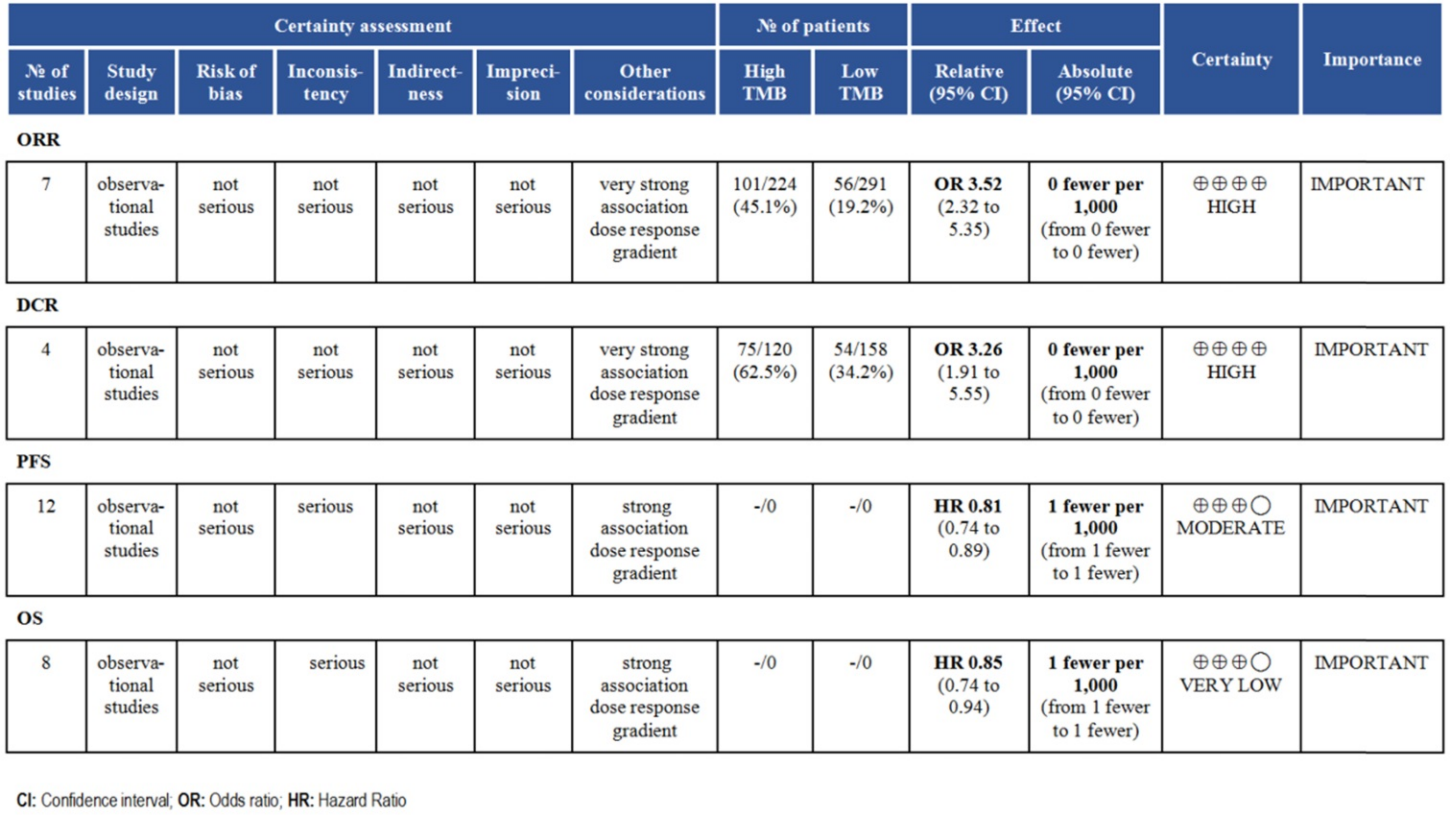
Summary of GRADE on evidences of outcomes.

According to the GRADE grading method, we analyzed the evidence level of each result, and the results showed that the evidence level of ORR and DCR was very high. For survival time, the evidence level of PFS and OS was moderate (Fig. 6).

## Discussion

Lung cancer is still the most common cause of cancer death worldwide, killing more than a million people each year 27. Most NSCLC patients are advanced at the time of diagnosis. In recent years, immunotherapy has been gradually applied to the therapy of NSCLC, and the selection of appropriate patients for immunotherapy is the focus of current therapy, and the selection of suitable patients with appropriate predictive indicators is the most important.

In previous studies, TMB was determined by whole exome sequencing (WES). WES can provide a comprehensive overview of protein-coding gene changes, but its high cost and time-consuming lead to limitations in routine practice 28,29. Although WES is still the standard method for quantifying TMB, there is growing evidence that targeted NGS panels with greater clinical value can also be used for sequencing TMB if sufficient genomic regions are covered 30. The US Food and Drug Administration (FDA) have approved Foundation One CDx and MSK-IMPACT for general clinical gene analysis. Although it has not yet been approved for these specific uses, most oncology platforms currently use targeted NGS for TMB sequencing 4,31.We included all studies that used targeted NGS as a TMB sequencing method and performed a meta-analysis.

First, we used ORR and DCR as the endpoints of our analysis to assess the effectiveness of ICI therapy. Hellman 32 howed that the ORR of NSCLC patients in the high TMB group for ICI therapy was superior to that in the low TMB group (*p* = 0.0005), ORR increased by 48%. Goodman 23 showed that the ORR of NSCLC patients in the high TMB group treated with ICI was superior to that in the low TMB group (*p* = 0.0077), ORR increased by 15%. Rizvi 12 showed that DCR of NSCLC patients treated with ICI in the high TMB group was superior to that in the low TMB group (*p* = 0.0011), DCR increased by 47%. We evaluated the relationship between TMB and the ORR and DCR of ICI therapy respectively. The results of meta-analysis were similar to the above results, indicating that the ORR and DCR of ICI therapy in the high TMB group were higher than those in the low TMB group, and there was no heterogeneity. In order to screen the predominant population for ICI therapy, we further performed a subgroup analysis. We performed group evaluations from the patient region and immunotherapy protocol. We found no matter regions (Asian and western people) or the immunotherapy protocols (anti-PD-1/PD-L1 combined with anti-CTLA 4 therapy and anti-PD-1/PD-L1 monotherapy), the ORR and DCR of ICI therapy in the high TMB groups were both higher than those in the low TMB groups. This suggests that we can screen patients for ICI therapy according to the level of TMB, which may have some significance for the advanced therapy of NSCLC.

In recent years, many studies have confirmed that ICI therapy can prolong PFS and OS in NSCLC patients 33,34,35, but some studies still hold the opposite view 15,20. In order to further screen suitable NSCLC patients for ICI therapy, we used the TMB threshold to judge the predictive ability of survival time of ICI therapy. Hellman 32 used WES method, and showed that the median PFS of high TMB group and low TMB group treated with ICI was 17.1 months versus 3.7 months (*p* = 0.0024), Alborelli 16 showed that the median PFS of high TMB group and low TMB group treated with ICI was 16.4 months versus 2.6 months (*p* = 0.0014), the median OS was 37.5 months versus 9 months (*p* = 0.0197), and Fang 22 showed that the median PFS of the high and low TMB groups treated with ICI was 4.3 months versus 2.0 months (*p* = 0.0018). However, Chae 20 showed that the PFS and OS of ICI therapy in low TMB group were both better than those in high TMB group. The results of this meta-analysis showed that patients with high TMB had superior PFS and OS in ICI therapy than those in low TMB, but the heterogeneity was obvious. Sensitivity analysis indicated stable results. In order to explore the source of heterogeneity and screen the dominant population, we further performed subgroup analysis. First, most of the studies included in this meta-analysis were conducted in western populations, and the meta-analysis results showed no significant difference between the PFS of ICI therapy in the high TMB group and the low TMB group. The analysis of Asian people showed that the PFS of ICI therapy in the high TMB group was better than that in the low TMB group, which may suggest that the TMB threshold may be suitable to screen the superior group of ICI therapy in the Asian people. In addition, we found that in the subgroup analysis of PFS, among the anti-PD-1/PD-L1 monotherapy group (*p* = 0.04) and anti-PD-1/PD-L1 combined with anti-CTLA-4 therapy group (*p* = 0.04), the PFS of ICI therapy in the high TMB group was superior to low TMB group. In the subgroup analysis of OS, OS of ICI therapy in patients with high TMB was superior to patients with low TMB in anti-PD-1/PD-L1 combined with anti-CTLA-4 therapy group (*p* = 0.04); and there was no difference in OS between the patients with high TMB and low TMB in anti-PD-1 / PD-L1 therapy group (*p* = 0.45). At present, there have been a lot of reports on anti-CTLA-4, anti-PD-1 and anti-PD-L1. Studies 36 have shown that in the tumor microenvironment, anti-CTLA-4 stimulates the activation of surrounding T cells by blocking the binding of CTLA-4 on the surface of T cells to the ligand CD80/CD86 on APCs, but does not activate T cells. Anti-PD-1/PD-L1 may play an anti-tumor effect by activating T lymphocytes. Although both are negative signals for T cell activation, their location and timing are different. CTLA-4 is expressed on T cells, while PD-1 is more widely expressed on a variety of cells. Normally, CTLA-4 suppresses T cells in the early stages of the immune cycle in lymph nodes, while PD-1 regulates the immune response in peripheral tissues or tumor sites 37,38. Pardoll's study demonstrated that anti-PD-1 targeting tumor infiltrating lymphocytes (TIL) can complement the anti-tumor activity of anti-CTLA-4 through non-redundant pathways 39.

In order to further verify our conclusion, we searched for an open access data platform-MSKCC through http://www.cbioportal.org. The database included 1662 patients with advanced cancer who were treated with ICI, of which 350 patients were diagnosed with NSCLC 40. We further obtained the TMB threshold histogram (sFigure 1) and survival curve (sFigure 2) of 350 patients. The results showed that patients with TMB threshold ≥12.27 have significantly better OS than patients with TMB threshold <12.27, which is consistent with our conclusion similar.

Finally, we analyzed the quality of the included studies and the level of evidence for outcome indicators discussed. NOS table indicated the relatively high quality of the included studies and improves the reliability of the meta-analysis. GRADE classification indicated the level of evidence for ORR and DCR was high, so it is reasonable to expect that the true effect is close to the estimated effect. For PFS and OS, we have limited confidence in the effect estimates and need to further enrich the included studies.

This meta-analysis is limited by several aspects. First of all, the sample sizes of the included studies are different, leading to large differences in sample sizes among different subgroups. Among them, the smaller the sample size, the more studies may be the main source of affecting the quality of the meta-analysis. Secondly, although the studies we included all adopted targeted NGS, the specific sequencing panel was different, which resulted in a certain fluctuation range of the TMB cut-off value and also affected the quality of meta-analysis to a certain extent. In addition, we lack data on comorbidities, ECOG scores, and previous treatment regimens for patients, which may play an important role in the effectiveness of ICI.

## Conclusions

TMB is a promising biomarker, which can predict the efficacy of ICI therapy in advanced NSCLC patients, included ORR, DCR, PFS and OS. Therefore, the advance measurement of TMB in clinical diagnosis can provide a basis for the treatment of patients with advanced NSCLC.

## Supplementary Material

Supplementary figures.

## Figures and Tables

**Table 1:** Main characteristics of studies included in the meta-analysis

Author	Time	Line of treatment	Sample size evaluable for TMB	Immunotherapy regimen	Area	TMB cut-off point	Outcomes
Rizvi	2018	≥1st-line	240	Anti-PD1/PD-L1	Western	10 Mut/Mb	PFS
Alborelli	2019	2nd-line	76	Anti-PD-1+Anti-CTLA4	Western	9 Mut/Mb	ORR, DCR, PFS, OS
Ready	2019	1st-line	98	Anti-PD-1+Anti-CTLA4	Western	10 Mut/Mb	ORR, DCR, PFS
Paz-ares	2018	1st-line	151	Anti-PD-1+Anti-CTLA4	Western	10 Mut/Mb	ORR
Wang	2019	≥1st-line	50	Anti-PD1/PD-L1	Asian	6 Mut/Mb	ORR, PFS
Chae	2018	≥1st-line	72	Anti-PD1/PD-L1	Western	15 Mut/Mb	PFS, OS
Chae	2019	≥1st-line	20	Anti-PD1/PD-L1	Western	Not given	PFS, OS
Gandara (POPLAR)	2018	≥2nd-line	105	Anti-PD-L1	Western	16 mutations	PFS, OS
Gandara (OAK)	2018	≥2nd-line	324	Anti-PD-L1	Western	16 mutations	PFS, OS
Fang	2019	N/A	75	Anti-PD-1/PD-L1	Asian	10 Mut/Mb	ORR, DCR, PFS
Reck	2019	N/A	1004	Anti-PD-1+Anti-CTLA4	Western	10 Mut/Mb	OS
Goodman	2017	≥1st-line	36	Anti-PD-1/PD-L1	Western	20 Mut/Mb	ORR, PFS, OS
Kowanetz	2017	1st-line	102	Anti-PD-1/PD-L1	Western	13.5 Mut/Mb	PFS, OS
Kowanetz	2017	≥2nd-line	371	Anti-PD-1/PD-L1	Western	17.1 Mut/Mb	PFS, OS
Roszik	2016	N/A	29	Anti-PD-1/PD-L1	Western	100 mutations	ORR, DCR
Genentech	2020	1st-line	119	Anti-PD-1/PD-L1	Western	16 Mut/Mb	PFS

Abbreviations: TMB: Tumor mutation burden; ORR: Objective response rate; DCR: Disease control rate; PFS: Progression-free survival; OS: Overall survival.

**Table 2:** Quality assessment of studies included in the meta-analysis using Newcastle Ottawa Scale (NOS)

Study	S1	S2	S3	S4	C	O1	O2	O3	Total score
rizvi2018	0	1	1	1	1	1	1	1	7
alborelli2019	1	1	1	1	2	1	1	1	9
ready2019	1	1	1	1	2	0	1	1	8
paz-ares2018	1	1	1	1	2	1	1	1	9
wang2019	1	1	1	1	2	1	1	1	9
chae2018	1	1	1	1	0	1	1	1	7
chae2019	1	1	1	1	0	1	1	1	7
gandara2018	1	1	1	1	1	1	1	1	8
fang2019	1	1	1	1	0	1	1	1	7
reck2019	1	1	1	1	0	1	1	1	7
goodman2017	1	1	1	1	0	1	1	1	7
kowanetz2017	1	1	1	1	0	1	1	1	7
roszik2016	1	1	1	1	1	1	1	1	7
genentech2020	1	1	1	1	0	0	1	1	6

S1: Representativeness of the exposed cohort; S2: Selection of the non-exposed cohort; S3: Ascertainment of exposure; C: Outcome of interest not present at start of study; O1: Comparability of cohorts; O2: Assessment of outcome; O3: Follow-up long enough; O4: Adequacy of follow up of cohorts.
