# Supplementary figures and images for: Predictive value of tumor mutation burden (TMB) with targeted next-generation sequencing in immunocheckpoint inhibitors for non-small cell lung cancer (NSCLC)
Source: J Cancer. 2021 Jan 1;12(2):584–94. doi: 10.7150/jca.48105 (PMC7738995; doi:10.7150/jca.48105)

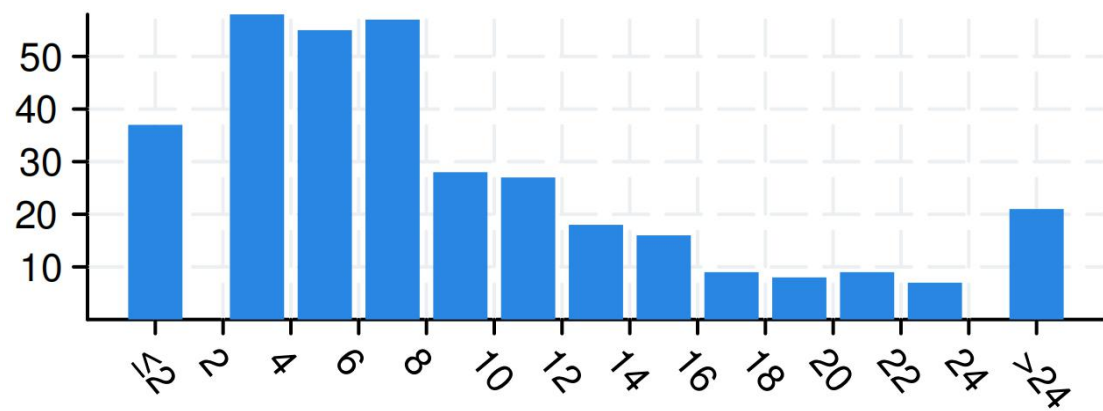

**sFigure 1.** TMB threshold histogram

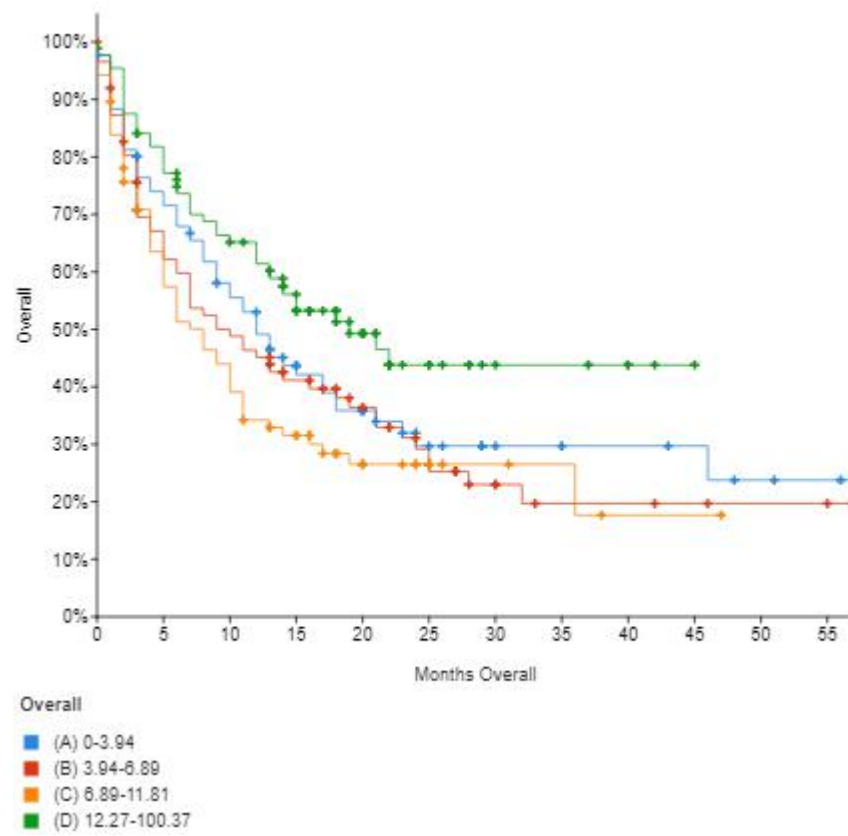

**sFigure 2.** Survival curves at different TMB thresholds

Supplement: Supplementary file 1 — Supplementary figures. [file jcav12p0584s1.pdf]
